# Comparative Biotypic and Phylogenetic Profiles of *Escherichia coli* Isolated from Resident Stool and Lagoon in Fresco (Côte d'Ivoire)

**DOI:** 10.1155/2019/9708494

**Published:** 2019-11-03

**Authors:** Nadége Kouadio-Ngbesso, Koua Atobla, Paul Yao Attien, Mireille Kouame-Sina, René Ahua Koffi, Antoinette Ama Adingra, Adjehi Dadié

**Affiliations:** ^1^Oceanology Research Center, BPV 18, Abidjan, Côte d'Ivoire; ^2^University Felix Houphouet Boigny, 01 BPV 34, Abidjan 01, Côte d'Ivoire; ^3^University Jean Lorougnon Guede of Daloa, BP 150, Daloa, Côte d'Ivoire; ^4^Pasteur Institute of Côte d'Ivoire, 01 BP 490, Abidjan 01, Côte d'Ivoire; ^5^Ecology Research Center, 08 BP 109, Abidjan 08, Côte d'Ivoire; ^6^University Nangui Abrogoua, 02 BP 801, Abidjan 02, Côte d'Ivoire

## Abstract

Anthropogenic activities could expose Fresco lagoon to microbial pollution. The objective of this study was to determine the level of pollution in Fresco lagoon related to fecal contaminations. Two hundred and seventy (270) samples including 216 water and 54 human stools samples from local residents were collected. *Escherichia coli* was isolated and identified according to classical bacteriology procedure. Strains were characterized by biotyping on API 20E gallery and phylogenetic typing by PCR triplex of Clermont. A set of 392 strains of *E. coli* was distributed into 18 biotypic profiles. Five biotypes were common to water and human. Classification of all biotypes revealed close relationship between water and human strains because of their repartition in the same groups. Phylogenetic groups A, B1, B2, and D were identified in all strains. Strains belonging to phylogenetic group A were most frequent in water (69.82%) and human stool (44.44%) followed by group B1 in water (24%) and human stool (40.7%). Strains of group B2 were scarce in water (4.4%) and humans (7.41%). The diversity of *E. coli* biotypes observed in this study revealed animal and human origins of contaminations. A close relationship was found between water and human strains, and the presence of commensal and extraintestinal pathogenic *E. coli* in all samples could represent a potential reservoir of extraintestinal infections for resident populations.

## 1. Introduction

*Escherichia coli* is a normal inhabitant of lower tract intestinal of warm-blooded animals and humans. However, in the past two decades, it has also been used as an indicator organism for source tracking purposes [1]; distribution of this organism has historically been used as an index of water quality [2]. Fresco lagoon, like many African coastal lagoons, is under consistent and sometimes severe pressure from diverse forms of human activities emanating from the surrounding city and villages. The indiscriminate dumping of untreated wastes into this aquatic environment could bring about physical, chemical, and biological deteriorations of water. This will no doubt endanger resident aquatic organisms as well as impair the beneficial uses of water. However, a paucity information exists on extent of biological pollution of Fresco lagoon and its public health implication. Contamination of water and food with fecal bacteria is and remains, a common and persistent problem, impacting public health and local and national economies [3]. *E. coli* is genotypically and phenotypically diverse. The role, the need, and the importance of biotyping bacterial isolates have been presented in various publications since 1970s [4–6]. Phenotypic characteristics are carbon utilization patterns, sugar use, antibiotic resistance profiles, flagellar motility, and other phenotypic characteristics. Phylogenetic studies showed that there are four main phylogenetic groups of *E. coli*, designated A, B1, B2, and D [7, 8]. Recent studies in 2013 revealed that there are eight recognized phylogroups of *E. coli* (A, B1, B2, C, D, E, F, and *Escherichia* clade I) [9], but this study realized in 2008-2009 and focused on the four main phylogroups. Extraintestinal pathogenic strains belong to groups B2 and D [10], whereas commensal strains to groups A and B1 [11]. Pathogenic strains have been associated with several diseases, including diarrhea, urinary tract infections, and meningitis [12]. In a recent study, *E. coli* belonging to phylogenetics groups A, B1, and B2 were isolated from fishes in Fresco lagoon [13]. The presence of *E. coli* represents a risk of disease if strains are found to be pathogenic. The aim of this present work was to study the relationship between *E. coli* strains, isolated from water of Fresco lagoon and resident stools, into biotype and phylogenetic groups and assess the likely source of contamination and the presence of potential pathogenic strains in Fresco lagoon.

## 2. Material and Methods

### 2.1. Water and Human Stool Sampling

**Figure 1:**
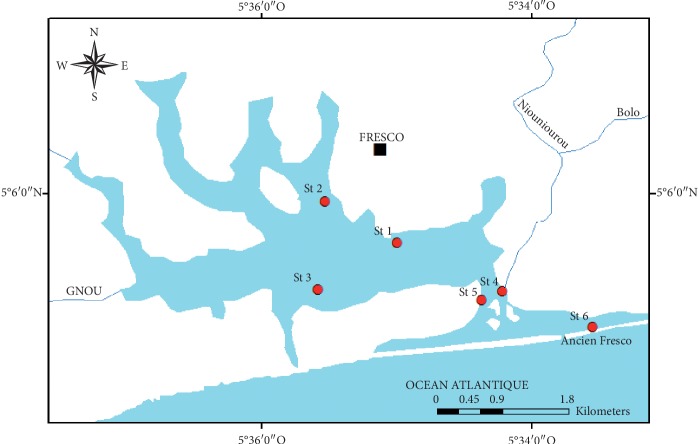
Map of Fresco lagoon showing water sampling sites. St: site.

A total of 216 surface water samples were included in this study. All samples were collected from Fresco lagoon, and the sampling sites were purposely selected from Fresco city and its surrounding areas based on the direct influence of sewage and the two rivers Niouniourou and Bolo. Sampling sites are shown in Figure 1. Water samples (250 ml) were collected at each site, 20 cm below the surface of lagoon using sterile glass bottles, stored on ice, and transported to laboratory. All samples were collected monthly from January 2008 to June 2009. For human stool, fifty-four samples were collected from local residents in general hospital of Fresco. *Escherichia coli* was identified using standard coproculture methods during June 2009. Petri dishes containing *E. coli* were conserved in stomacher bags on ice and transported to microbiology laboratory of Oceanology Research Center for analyzing before 24 h.

### 2.2. *E. coli* Isolation, Identification, and Biotyping

According to membrane filter technique for quantification of fecal coliform [14], 100 ml of water was filtered and placed directly on EMB agar medium (Merck, France). For human stool, *E. coli* strains were inoculated onto EMB agar medium. Plates were incubated at 37°C for 24 h. After incubation, presumptive *E. coli* (purple with metallic sheen) were counted. Strains were selected and inoculated onto nutrient agar (Bio-Rad) before incubation at 37°C for 24 h to obtain pure cultures for further analysis. Pure cultures after Gram coloration were tested for cytochrome oxidase and catalase production.

For biotyping, the method used was to establish a biochemical profile of strains based on the traits highlighted with an API 20E gallery [6, 15]. The gallery (Biomerieux SA, France) was used for control diagnosis for the identification of the species on one hand and the analysis of the results of the test, taking into account the variant or nonconstant biochemical characters, served as a discriminating factor of one strain to another for the determination of biotypes.

Stock cultures were stored in brain heart infusion broth (BHI) (Difco, Michigan, USA) with 20% glycerol at −80°C until use.

### 2.3. DNA Extraction

*E. coli* isolates were grown in Luria-Bertani (LB) broth at 37°C overnight. Bacteria from 1.5 ml of growth medium were pelleted by centrifugation at 1200 g for 10 min. Bacterial pellet was suspended in 200 *μ*l of sterile ultrapure water and boiled for 10 min in a water bath (100°C). The lysate was centrifuged again, and supernatant was used directly as the template for PCR.

### 2.4. Phylogenetic Group Determination

The phylogenetic group of each strain isolated was determined by triplex PCR [7]. For a reaction mixture of 25 *μ*l, 2.5 *μ*l of 10X buffer (Q biogenic, France), 0.25 ml of each 20 pmol of primer concentration (Eurofins, Germany), 4 *μ*l of 2 *μ*M DNTP (MP, France), 0.25 *μ*l of Taq polymerase at a concentration of 2.5 U (Biolabs, France), 1.5 *μ*l of 25 mM MgCl_2_ (MP, France), and 5 *μ*l of DNA extract were mixed. Mixture was made up to a final reaction volume of 25 *μ*l with water. Amplification was achieved in thermocycler Biometra Uno II, Biotron 1998, (Germany) according to following program: an initial denaturation step at 94°C for 5 min, followed by 30 cycles composed of denaturation at 94°C for 30 s, hybridisations at 55°C for 30 s, and extension at 72°C for 30 s. After these cycles, a final extension at 72°C for 7 min is realised. Separation of amplification products were performed by agarose gel electrophoresis. Migration was performed in 0.5X TBE at 100 V for 40 min. After migration, DNA bands were visualized by fluorescence under UV at 360 nm and photographed using an Uvitec camera (United Kingdom). And then, phylogenetic groups were determined using dichotomous tree of Clermont et al. [7].

### 2.5. Statistical Analysis

Statistical analyses were carried out using SPSS 20.0 software. Hierarchical classification analysis was used to group biotypes based on similar biochemical characters using this software. Results were represented by dendrogram. Euclidean distance was used in this analysis, and the Ward method was used as an aggregation criterion.

## 3. Results

### 3.1. Abundance of *E. coli* in Water and Isolation Rates in Human Stool

**Figure 2:**
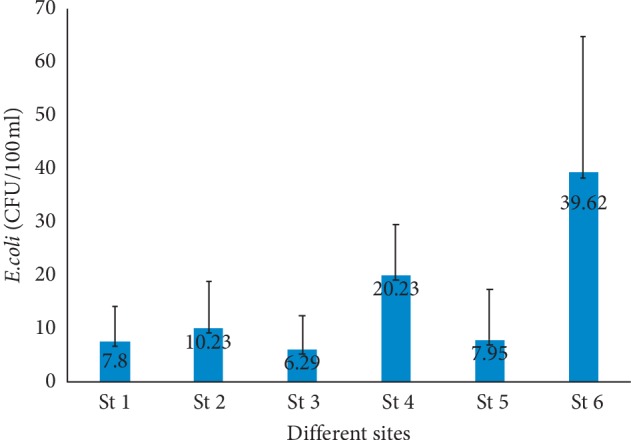
Abundance of *E. coli* in the different sampling sites of Fresco lagoon.

*Escherichia coli* were detected in all water samples (216), in all sites, and 54 human stool were analyzed. The mean (from January 2008 to June 2009) *E. coli* count varied from 6.29 to 39.62 CFU (colony forming unit) in 100 ml of water analyzed (Figure 2). For human stool, fifty-four strains of presumptive *E. coli* (one for each sample) were isolated for further analysis.

### 3.2. Biotype of *E. coli* Identified in Water and Human Stool

Eighteen biotypes of *E. coli* were observed in water and human strains (338 and 54, respectively) (Table 1). The different biotypes obtained are compared to biotype 1 (B1), which according to Biomerieux corresponds to the reference strain *Escherichia coli* ATCC 25922. Differences were based on the presence or lack of biochemicals characteristics such as ADH (arginine dihydrolase), LDC (lysine decarboxylase), ODC (ornithine decarboxylase), INO (inositol), SOR (sorbitol), RHA (rhamnose), SAC (saccharose), and MEL (melibiose). Twelve biotypes were identified in water strains and eleven in human stool. Five biotypes were common to strains of water and human stool (B1, B2, B3, B6, and B8). Biotypes 1 and 6 are frequently encountered with respective frequencies of 20.11% and 61.24% in water and 22.22% and 29.62% in human stool samples. Biotypes 15, 16, 17, 18, and 19 were specific for human stool in this study. Biotypes 4, 5, 7, 9, 10, 11, and 14 were isolated only in water at 0.6% to 2.36% (Table 2). Biotype B12 was not identified in water and human stool samples, but only in fish strains (published in previous study). For reasons of coherence with the preceding articles, no change has been made in the numbering of biotypes.

### 3.3. Relationship between Human and Water Biotypes

**Figure 3:**
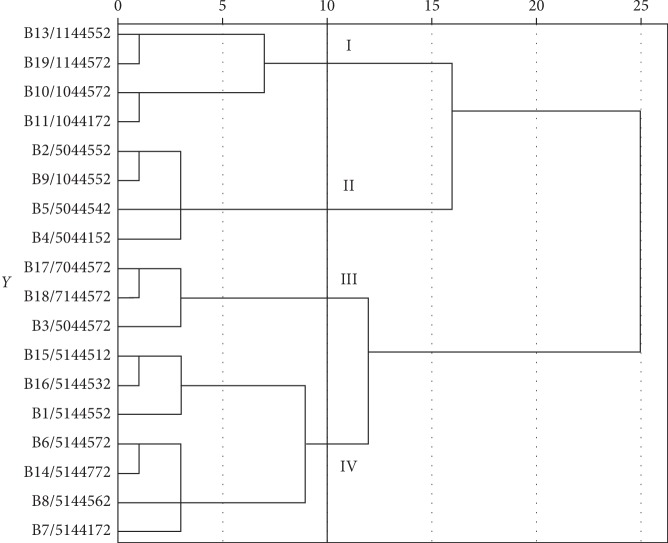
Dendrogram revealing the relationship between human and water biotypes.

Clustering analysis to determine the relationship between human and water biotypes using the results of biochemical tests was performed. From the distance of Ward 10, all 18 biotypes are divided into 4 groups (Figure 3). In groups I, II, III, and IV, there were both human and water biotypes. There was no specific group to human or water. There was a relationship between all biotypes identified in this study.

### 3.4. Phylogenetic Groups of Strains Isolated from Water and Human Stool

**Figure 4:**
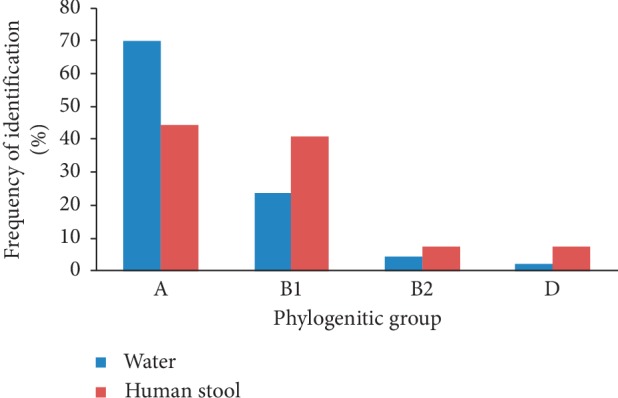
Distribution of phylogenetic groups of *E. coli* in water and human stool (%).

Dichotomous decision tree used to determine phylogenetic groups of *E. coli* strain revealed the presence of groups A, B1, B2, and D in water and human strains. Of 338 strains of *E. coli* isolated in water from Fresco lagoon, strains of group A were most common (69.82%), followed by strains of group B1 (23.67%). Strains of groups B2 and D were rare (4.44% and 2.07%, respectively). Of 54 strains recovered from human stool, phylogenetic groups A, B1, B2, and D were found too. Strains mostly isolated were from groups A and B1 at 44.44% and 40.74%, respectively. Groups B2 and D totalized only 7.41% of all analyzed strains (Figure 4).

## 4. Discussion

*Escherichia coli* is used worldwide as a fecal indicator species to assess the water quality in aquatic environments [16, 17]. The presence of *E. coli* at all sites is an evidence of fecal contamination throughout the lagoon. In fact, local residents' unhygienic habits at the lagoon induce this contamination. The sites ST6 (formerly Fresco), ST1 (boat zone), and ST2 (behind the farm), marked by human and animal presence, are distinguished with a high load of *E. coli*. The identification of eighteen different biotypes in water and human stool analyzed revealed the presence and great diversity of *E. coli* in lagoon the environment. Biotype 1 according to BioMerieux corresponds to reference *Escherichia coli* ATCC 25922. Biotype 6 with the highest frequency of isolation both in water and human strains (61.24% and 29.62%, respectively) had a similar profile with the strain isolated in North Carolina, which was responsible for enteritis and Turkey mortality syndrome [18]. In addition, in this biotype, the production of colicine has been shown, which is associated with the virulence of avian *E. coli* [19]. Biotypes 1, 2, and 6 correspond to those isolated during urinary *E. coli* infections in patients [20]. According to Swansson and Collins [21], the same biotypes (1, 2, and 6) were, as in this study, most frequently isolated among veterinary strains. Biotypes 3, 5, and 9 isolated in water and human stool have also been isolated from veterinary strains [21]. Biotype 7 isolated only in water has also been identified according to Nijsten et al. [22] in faeces of breeders pigs and according to Loukiadis [23] in pretreated effluents from slaughterhouses in France. Biotypes 4, 7, and 11 isolated only in water are negative for sorbitol, one of the biochemical characteristics used in screening of *E. coli* O157 : H7 (EHEC) which is responsible of several pathologies including, hemorrhagic colitis, hemolytic, and uremic syndrome [24]. These biotypes were specific to sampling sites. Biotype 4 was specific to site 4, where rivers Bolo and Niouniourou join and flow into the lagoon. Biotypes 7 and 11 were specific to site 6, which corresponds to the old village of Fresco, where fishermen and their families live. They have pigs, poultry, and sheep. In this site, there is no sanitation system and high level of *E. coli* (39.62 CFU/100 ml) was detected there. Biotypes 4, 10, 11, and 14 isolated in water and biotype 8 isolated in water and human stool have also been identified in pretreated effluents and sludge collected in slaughterhouses in France [23]. Biotype 8 was isolated in water at site 2, where there is presence of fishing nets and fishermen and not far from some homes where there are poultry farms and sheep. These diverse phenotypes observed result principally from a large number of different gene combinations [25] and could be the fact that strains of animals or humans' origins are released into the environment without treatment and resident populations use the lagoon as a waste receptacle (stool and effluents). In addition, the presence of pigs and chicken farms near water can also explain bioburden. Indeed, the waste or droppings emanating from these farms are either dumped directly into the lagoon without treatment or drained in the environment by leaching caused by runoff water in rainy season. Moreover, some villages around the lagoon have no sanitation system. Hierarchical classification analysis of all biotypes has shown close relationship between water and human strains. It was not possible to distinguish between strains of human and animal origin [25]. Human and animal strains causing the same disease in different hosts share a common pool of virulence genes [25].

Since contamination from *E. coli* represents a risk for human health, gathering more information about *E. coli* strains isolated from water and human stool samples by assigning them to the phylogenetic groups A, B1, B2, and D using the PCR technique described by Clermont et al. [7] was necessary. As expected, the four phylogenetic groups were found both in strains of water and human stool. Strains belonging to groups A and B1 are mostly distributed in water (75%) and human stool (85%). Duriez et al. [26] found that in 168 nonepidemiological-related isolates from three geographically distinct human populations, strains from phylogenetic groups A and B1 were the most common; strains of group B2 were rare. *E. coli* strains are usually referred as commensal, intestinal, or extraintestinal pathogenic [27]. Extraintestinal pathogenic strains belong to groups B2 and D [7, 10, 25], whereas the commensal strains to groups A and B1 [11, 25]. Our study demonstrated that commensal strains are most abundant in water of Fresco lagoon. Strains belonging to groups B2 and D are less represented in water and human stool samples. These results are in agreement with those of Walk et al. [28]. Picard et al. [29] found that B2 strains accounted for only 9% of examined commensal human strains. *E. coli* strains from group B2 are highly pathogenic and frequently responsible for extraintestinal infections [26, 30, 31] and for urinary tract infections in humans [32]. Extraintestinal pathogenic *E. coli* can be found in group D [30], and according to Clermont et al. [7], *E. coli* O157 : H7 could belong to this group. The presence of groups B2 and D in the water might suggest that Fresco lagoon can present a risk of extraintestinal pathogenic E. coli for inhabitants who use this water for recreational activities.

## 5. Conclusion

The diversity of *E. coli* biotypes observed in this study revealed animal and human origins of contaminations. A close relationship was found between water and human strains. The presence of commensal and extraintestinal pathogenic *E. coli* in all samples could represent a potential reservoir of extraintestinal infections for resident populations.

## Figures and Tables

**Table 1:** Biotype of *E. coli* identified in water and human stool based on differences between biochemicals characteristics. B = biotype; ADH: arginine dihydrolase; LDC: lysine decarboxylase; ODC: ornithine decarboxylase; INO: inositol; SOR: sorbitol; RHA: rhamnose; SAC: saccharose; MEL: melibiose; −: negative for reaction; +: positive for reaction. Reference strain: B1.

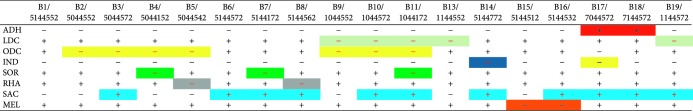

**Table 2:** Repartition of biotypes in water and human stool.

	Water (%)	Human stool (%)
B1	20.11	22.22
B2	2.96	11.11
B3	5.63	7.40
B4	1.18	0
B5	1.18	0
B6	61.24	29.62
B7	1.18	0
B8	2.36	3.71
B9	2.07	0
B10	0.6	0
B11	0.89	0
B13	0	3.71
B14	0.6	0
B15	0	7.40
B16	0	3.71
B17	0	3.71
B18	0	3.71
B19	0	3.71

B: biotype.

## Data Availability

The data used to support the findings of this study are available from the corresponding author upon request.
